# Transcriptome Analysis Revealed Overlapping and Special Regulatory Roles of RpoN1 and RpoN2 in Motility, Virulence, and Growth of *Xanthomonas oryzae* pv. *oryzae*

**DOI:** 10.3389/fmicb.2021.653354

**Published:** 2021-03-04

**Authors:** Chao Yu, Doan-Phuong Nguyen, Fenghuan Yang, Jia Shi, Yiming Wei, Fang Tian, Xiuxiang Zhao, Huamin Chen

**Affiliations:** ^1^State Key Laboratory for Biology of Plant Diseases and Insect Pests, Institute of Plant Protection, Chinese Academy of Agricultural Sciences, Beijing, China; ^2^College of Plant Protection, Shenyang Agricultural University, Shenyang, China

**Keywords:** σ^54^ factor, *Xanthomonas oryzae* pv. *oryzae*, transcriptome analysis, motility, virulence, growth

## Abstract

σ^54^ factor (RpoN) plays a crucial role in bacterial motility, virulence, growth, and other biological functions. In our previous study, two homologous σ^54^ factors, RpoN1 and RpoN2, were identified in *Xanthomonas oryzae* pv. *oryzae* (*Xoo*), the causative agent of bacterial leaf blight in rice. However, their functional roles, i.e., whether they exert combined or independent effects, remain unknown. In the current study, *rpoN1* or *rpoN2* deletion in *Xoo* significantly disrupted bacterial swimming motility, flagellar assembly, and virulence. Transcriptome analysis led to the identification of 127 overlapping differentially expressed genes (DEGs) regulated by both RpoN1 and RpoN2. Furthermore, GO and KEGG classification demonstrated that these DEGs were highly enriched in flagellar assembly, chemotaxis, and c-di-GMP pathways. Interestingly, *ropN1* deletion decreased *ropN2* transcription, while *rpoN2* deletion did not affect *ropN1* transcription. No interaction between the *rpoN2* promoter and RpoN1 was detected, suggesting that RpoN1 indirectly regulates *rpoN2* transcription. In addition, RpoN1-regulated DEGs were specially enriched in ribosome, carbon, and nitrogen metabolism pathways. Besides, bacterial growth was remarkably repressed in Δ*rpoN1* but not in Δ*rpoN2*. Taken together, this study demonstrates the overlapping and unique regulatory roles of RpoN1 and RpoN2 in motility, virulence, growth and provides new insights into the regulatory mechanism of σ^54^ factors in *Xoo*.

## Introduction

Transcription regulates a myriad of biological processes. In bacteria, σ factors are the most widely occurring transcriptional regulators that reversibly bind RNA polymerase (RNAP) and regulate transcription of numerous functional genes. σ factors initiate the RNA synthesis process by guiding RNAP holoenzyme binding to specific promoters, melting double-stranded promoter DNA strands, and stabilizing it as a single-stranded open complex. Till now, only two families of σ factors, i.e., σ^70^ and σ^54^, have been reported (Kazmierczak et al., 2005). Unlike the major σ^70^ class, σ^54^ factors interact with other transcriptional activators, as also named enhancer-binding proteins (EBPs), to induce gene expression (Studholme and Dixon, 2003; Davis et al., 2017). Furthermore, σ^54^ factor binding sites are typically located at the −24/−12 region of the highly conserved sequence, i.e., GGN_10_GC, whereas that of σ^70^ is located at the −35/−10 region (Barrios et al., 1999; Yang et al., 2015). Due to the indispensable role of σ^54^ factor in the transcription process, it is essential to identify its target genes and discern its regulatory pathways.

σ^54^ factor, alternatively named as RpoN, has been implicated as a multifunctional regulator of multiple vital biological processes, such as nitrogen assimilation, swimming motility, biofilm formation, extracellular polysaccharides (EPS) production, virulence, and type III secretion system (Kustu et al., 1989; Yang et al., 2009; Hao et al., 2013; Lee et al., 2016). Recent studies have focused on RpoN as a major regulator of bacterial growth, carbohydrate metabolism, twitching motility, quorum sensing, and type VI secretion system (Cai et al., 2015; Hayrapetyan et al., 2015; Ray et al., 2015; Shao et al., 2018). In addition, RpoN has been associated with environmental adaptation and tobramycin resistance (Viducic et al., 2017; Xu et al., 2019). Thus, it is necessary to thoroughly understand the complex regulatory network of bacterial RpoN.

The *rpoN* gene is widely distributed in bacteria. The majority of bacterial species contain only one copy of the *rpoN* gene; however, certain bacterial species contain two or more copies of the *rpoN* gene. Four *rpoN* genes were identified in *Rhodobacter sphaeroides* with distinct biological functions (Poggio et al., 2002). Two functionally distinct σ^54^ factors, i.e., RpoN1 and RpoN2, were studied in *Ralstonia solanacearum*. The outcomes showed that only RpoN1 but not RpoN2 was required for virulence, twitching motility, natural competence, and growth (Lundgren et al., 2015; Ray et al., 2015). In *Xanthomonas campestris*, σ^54^ factor RpoN2 regulates flagellar biosynthesis, swimming motility, biofilm formation, EPS, and virulence. In contrast, its homologous protein RpoN1 is involved in branched-chain fatty acid production and quorum sensing (Li et al., 2020). These studies demonstrated specific regulatory roles and biological functions of σ^54^ factors in pathogenic bacteria.

*Xanthomonas oryzae* pv. *oryzae* (*Xoo*), a gram-negative bacterium and causative agent of bacterial blight, leads to severe economic loss in the field. Besides, it is used as an ideal model to study the molecular mechanisms of bacterial pathogenicity in monocot plants (Nino-Liu et al., 2006). *Xoo* contains several genes related to motility, chemotaxis, EPS, biofilm, and cyclic dimeric guanosine monophosphate (c-di-GMP), controlled by diverse transcriptional factors (Das et al., 2009; White and Yang, 2009; Yang et al., 2019). Two σ^54^ factors, RpoN1 and RpoN2, were identified in *Xoo* in our previous work (Tian et al., 2015). The *rpoN2* gene is located in the flagellar regulon and transcribed in an operon with *fleQ*, encoding one of σ^54^ EBPs. RpoN2 controls flagellar movements and motility by regulating gene expression and virulence through an unknown mechanism (Tian et al., 2015). In addition, RpoN2 and FleQ regulate the post-translational modification of flagellin (Yu et al., 2018a). However, the unique biological functions of RpoN1 and overlapping functions of RpoN1 and RpoN2 in *Xoo* remain unclear.

In this study, RNA sequencing (RNA-seq) was employed to discern the regulatory networks and functional roles of RpoN1 and RpoN2 in *Xoo* swimming motility, virulence, and growth. Unlike σ^54^ factors in *Ralstonia solanacearum* and *Xanthomonas campestris*, in *Xoo*, RpoN1 and RpoN2 play important regulatory roles in flagellar assembly, swimming motility, chemotaxis, and c-di-GMP signal system. The results of this study will extend our understanding of the biological functions and regulatory mechanisms of bacterial σ^54^ factors.

## Materials and Methods

### Bacterial Strains, Plasmids, and Growth Conditions

Details of the bacterial strains and plasmids employed in this study are listed in Supplementary Table 1. *Xoo* strains were cultured in M210 liquid medium (0.8% casein enzymatic hydrolyzate, 0.4% yeast extract, 0.5% sucrose, 17.2 mM K_2_HPO_4_, 1.2 mM MgSO_4_⋅7H_2_O), PSA medium (1% tryptone, 0.5% sucrose, 0.1% glutamate, and 1.5% agar, pH 7.0), and XOM2 medium [0.18% D-(+) xylose, 14.7 mM KH_2_PO_4_, 10 mM sodium L-(+) glutamate, 5 mM MgCl_2_, 670 μM L-methionine, 240 μM Fe(III)-EDTA, and 40 μM MnSO_4_, pH 6.5] and incubated at 28°C for 24 h. *Escherichia coli* strains were cultured in Luria-Bertani medium (1% peptone, 1% NaCl, and 0.5% yeast extract) and incubated at 37°C for 24 h. The antibiotics ampicillin (Ap) and kanamycin (Km) were used at the concentration of 100 and 50 μg/mL, respectively.

### Construction of Mutants

Mutants derived from *Xoo* MAFF 311018 strain were generated by homologous recombination, as described previously (Yu et al., 2018a). Concisely, to amplify the left and right arms of *rpoN1*, *fliC*, *fliD*, *fliS*, *fleQ*, *fliA*, and *flgRR* genes, *Xoo* genomic DNA was subjected to PCR amplification using relevant F/R primers (Supplementary Table 2). The amplified PCR products were ligated into suicide vector pKMS1, and resulting plasmid vectors were referred to as pKM-*rpoN1*, pKM-*fliC*, pKM-*fliD*, pKM-*fliS*, pKM-*fleQ*, pKM-*fliA*, and pKM-*flgRR*. Later, these cloned plasmid vectors were electrophoretically transformed into *Xoo*. The transformants were first selected using NAN agar (1% tryptone, 0.3% peptone, 0.1% yeast extract, and 1.5% agar) containing Km, followed by continuous transfer culture in NBN broth (NAN without agar) for three times. The candidates were screened on NAS medium (NBN plus 10% sucrose). The Km sensitive mutants that grew on NAS medium were identified by PCR analysis. To generate the double mutant of *rpoN1* and *rpoN2*, the pKM-*rpoN1* plasmids were transformed into Δ*rpoN2* through electroporation method, and double mutants were screened as mentioned above. To obtain the complemented strains, the full-length of *rpoN1* and *rpoN2* were amplified using appropriate F/R primers (Supplementary Table 2) and ligated into the pBBR1MCS-4 vector. The resulting recombinant plasmids, pBBR-*rpoN1*, and pBBR-*rpoN2*, were electroporated into the relevant mutants. Lastly, the complemented strains were validated using PCR.

### RNA Extraction

*Xoo* cells were cultured in M210 medium at 28°C till the culture reached the optical density of 1.0 at 600 nm (OD_600_). The bacterial culture was centrifuged at 8,000 × *g* for 5 min. The bacterial cell pellet was resuspended in an equal volume of XOM2 medium and cultured at 28°C for 1 h. The bacterial medium was again centrifuged at 12,000 × *g* for 5 min to harvest bacterial cells. Total RNA was isolated from resulting bacterial cells using an RNAprep Pure Bacteria Kit (Tiangen, Beijing, China), and total RNA was stored at −80°C for RNA sequencing.

### RNA Sequencing and Data Analysis

RNA sequencing was performed by Novogene (Novogene, Beijing, China). The sequencing libraries were generated using NEBNext^®^ Ultra^TM^ Directional RNA Library Prep Kit for Illumina^®^ (New England Biolabs, Ipswich, MA, United States), as described previously (Fan et al., 2019) and as per manufacturer’s instructions. To assign sequences to each sample, the index codes were added. The clustering of the index-coded samples was performed on a cBot Cluster Generation System using TruSeq PE Cluster Kit v3-cBot-HS. The resulting library was sequenced on an Illumina Hiseq platform to generate paired-end reads.

Raw data in the FASTQ format were processed using in-house Perl scripts. To obtain clean reads, reads containing adapter or ploy-N, and low-quality reads were removed. Bowtie 2-2.2.3 was used to align clean reads to the *Xoo* reference genome (Langmead and Salzberg, 2012), and HTSeq version 0.6.1 was used to count the number of reads mapped to each gene. The expected number of FPKM (Fragments Per Kilobase of transcript sequence per Millions base pairs sequenced) of each gene was calculated based on the length of the gene and read count mapped to the gene (Trapnell et al., 2009). The RNA-Seq data were deposited in NCBI Sequence Read Archive (SRA) with the SRA Series accession number: PRJNA684983. DESeq R package version 1.18.0 was employed to identify differentially expressed genes in two samples. The threshold with *P* ≤ 0.05 and the absolute value of log_2_Ratio ≥ 1 was used to determine the significance of differential gene expression.

### Functional Analysis of Differentially Expressed Genes (DEGs)

GOseq R package^1^ was employed for the Gene Ontology (GO) enrichment analysis of DEGs. The GO terms with corrected *p*-value < 0.05 were considered significantly enriched. Kyoto Encyclopedia of Genes and Genomes (KEGG) analysis was performed as described previously (Mao et al., 2005), and KOBAS software was used to determine the significant enrichment of DEGs in KEGG pathways.

### Quantitative Real-Time PCR (qRT-PCR) Assay

Bacterial strains were cultured, and cells were collected as mentioned above. Total RNA was extracted from bacterial cells using the RNAprep pure Cell/Bacteria Kit (Tiangen Biotech), and cDNA was synthesized using HiScript II RT SuperMix kit (Vazyme, Nanjing, China). The qRT-PCR primers were designed using Primer Premier version 5.0 software (PREMIER Biosoft, Palo Alto, CA, United States) (Supplementary Table 2) with the *gyrB* as a reference gene. Finally, qRT-PCR was performed as described previously (Yu et al., 2020).

### Swimming Motility Assay

*Xoo* strains were cultured, as mentioned above. The bacterial medium was centrifuged at 12,000 × *g*, resuspended in distilled water, and OD_600_ was adjusted to 0.8. 2 μl of bacterial suspension was inoculated onto semisolid medium plates (0.03% peptone, 0.03% yeast extract, and 0.25% agar) and incubated at 28°C. The swimming zones of these bacteria were evaluated after 4 days. The assays were repeated three times.

### Electron Microscopy Visualization

The flagella of *Xoo* strains were visualized as described previously (Yu et al., 2018a). Briefly, *Xoo* strains were cultured on PSA plates at 28°C for 48 h, suspended in distilled water, and the bacterial suspension was transferred to Formvar coated grids (Standard Technology, Ormond Beach, FL, United States). The bacterial cells were stained with 2% uranyl acetate for 30 s, dried at room temperature, and visualized using the transmission electron microscope (H-7500, Hitachi, Tokyo, Japan).

### Virulence Assay

*Xoo* inoculation and bacterial growth assay were performed as described previously (Yu et al., 2018b). Briefly, *Xoo* strains were cultured overnight in M210 medium, and the bacterial medium was centrifuged at 7,000 × *g*, resuspended in distilled water, and OD_600_ was adjusted to 1.0. The *Xoo* strain suspension was applied on susceptible rice (*Oryza sativa* L. ssp. *Indica* IR24) by leaf clipping method, and the lesion lengths of 10 leaves were measured at 2 weeks after inoculation for every strain. For bacterial population assay, three *Xoo* strain inoculated rice leaves were weighed and ground with distilled water followed by its dilution to attain specific optical concentration, and later spread onto PSA plates. The *Xoo* colonies were counted after 72 h incubation. These experiments were performed three times independently.

### Electrophoretic Mobility Shift Assay (EMSA)

The DNA fragment of *rpoN1* was amplified with specific primer pairs N1F/R, and the amplified PCR product was cloned into pColdSUMO, resulting in plasmid pC-*rpoN1*. This recombinant plasmid was transformed into *E. coli* BL21 strain. RpoN1 protein expression and purification were performed as described previously (Yu et al., 2020). For EMSA assay, *rpoN2*, and *glnA* promoters (−300 to +50 upstream or downstream of the translation start [+1]) were PCR amplified using 5′ end FAM-labeled primers. 2 μM of labeled *rpoN2* or *glnA* promoter, 5 μM RpoN1 protein, and 1X EMSA/GelShift Binding Buffer (Beyotime, Shanghai, China) were mixed, and resolved using 4% native (wt/vol) polyacrylamide gel and electrophoresed for approximately 1 h at 100 V. The outcome of electrophoresis was detected on Typhoon FLA-5100 (Fuji Film, Tokyo, Japan) at 488 nm. All primers used in this experiment are listed in Supplementary Table 2.

### *In vitro* Growth Rate Measurement

*Xoo* strains were inoculated into M210 medium, and bacterial cell concentration was adjusted to obtain OD_600_ of 0.05 and incubated at 28°C under 200 rpm. The bacterial population was measured after every 6 h. For bacterial population assay, the bacterial cells were spread onto PSA plats after optional diluted, and cultured at 28°C for 3 days, the bacterial colonies then were counted. These experiments were repeated three times independently.

### Statistical Analysis

Swimming zones, disease lesion length, bacterial growth measurement, and relative gene expression were presented as means ± standard deviation. Two-tail Student’s *t*-test was performed with statistical significance set to 0.05 confidence level.

## Results

### Two σ^54^ Factors in *Xoo*

To elucidate the function of σ^54^ factor in *Xoo*, the protein sequence of *Xoo* σ^54^ factors was aligned against *Pseudomonas syringae* pv. *tomato* DC3000 (*Pst*) and *X*. *campestris* pv. *campestris* (*Xcc*) σ^54^ factor (RpoN) protein sequences. The outcomes indicated that RpoN1_Xoo_ protein shared 41, 94, and 40% identical residues with RpoN_Pst_, RpoN1_Xcc_, and RpoN2_Xcc_, respectively (Supplementary Figure 1). RpoN2_Xoo_ protein sequence was aligned against protein sequence of RpoN_Pst_, RpoN1_Xcc_, RpoN2_Xcc_, and it showed 39, 40, and 93% sequence similarity, respectively (Supplementary Figure 1). Interestingly, RpoN1_Xoo_ protein shared only 39% identical residues with RpoN2_Xoo_, but both contained conserved σ^54^ factor domains, including activator interacting domain (AID) and DNA binding domain (DBD) (Supplementary Figure 1).

### RpoN1 and RpoN2 Positively Regulate Swimming Motility and Virulence

**FIGURE 1:**
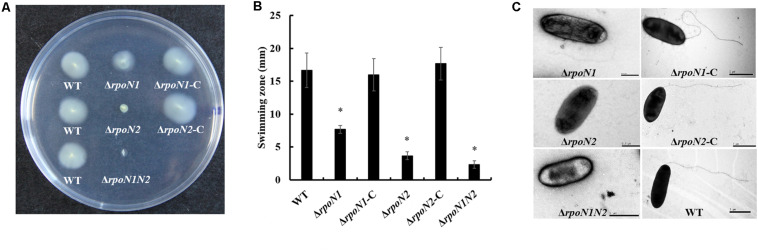
RpoN1 and RpoN2 for swimming motility and flagellar assembly in *Xanthomonas oryzae* pv. *oryzae* (*Xoo*). **(A)** Schematic representation and **(B)** quantitative analysis of swimming motility assay. The values represent the average of three independent experiments. Asterisks indicate significant differences between wild-type and mutants (*P* < 0.05) (Student’s *t* test). **(C)** Visualization of flagellar filament using scanning electron microscopy. WT, wild-type; Δ*rpoN1*, *rpoN1* gene deletion mutant; Δ*rpoN1*-C, the complemented strain of Δ*rpoN1*; Δ*rpoN2*, *rpoN2* gene deletion mutant; Δ*rpoN2*-C, the complemented strain of Δ*rpoN2*; Δ*rpoN1N2*, double gene (*rpoN1* and *rpoN2*) deletion mutant.

**FIGURE 2:**
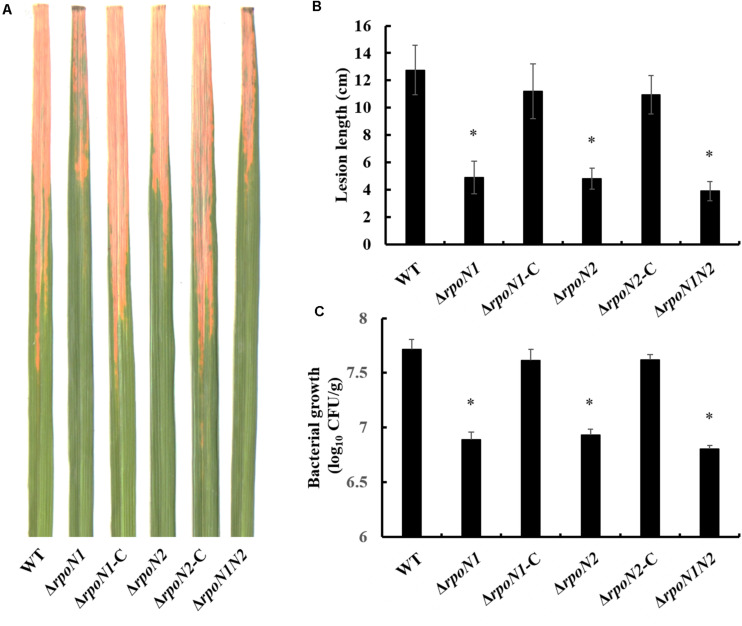
RpoN1 and RpoN2 positively regulate the virulence in *Xoo*. **(A)** Disease symptoms, **(B)** disease lesion lengths, and **(C)** bacterial population. Disease symptoms and lesion lengths were photographed and measured in the second week after inoculation. For bacterial population assay, three leaves were collected and mixed as one sample for each strain. The experiments were performed three times independently. Asterisks indicate significant differences between wild-type and mutants (*P*-value < 0.05) (Student’s *t* test).

σ^54^ factors affect the flagellar assembly and virulence by regulating the transcription of multiple genes (Yang et al., 2009; Ray et al., 2015). In this study, the gene knockout strains Δ*rpoN1* and Δ*rpoN2* and the double-mutant strain Δ*rpoN1N2* were constructed through homologous recombination to investigate the regulatory roles of RpoN1 and RpoN2 on *Xoo’* motility and virulence. Firstly, the swimming zones of mutated *Xoo* strains on semisolid medium plates were measured. As the result showed, swimming motility of Δ*rpoN1* was significantly reduced, and that of Δ*rpoN2*, Δ*rpoN1N2* was nearly lost compared to the wild-type strain (Figures 1A,B). Later, flagella of these strains were visualized using the scanning electron microscope. As speculated, Δ*rpoN1* strain showed abnormal flagella, but flagella were absent in Δ*rpoN2* and Δ*rpoN1N2* (Figure 1C). In addition, the swimming motility and flagellum synthesis of these mutants were restored in the relevant complemented strains (Figure 1). The results suggested that RpoN1 and RpoN2 positively regulated *Xoo* swimming motility by regulating the flagellar assembly. Finally, the virulence of these *Xoo* strains for the susceptible rice was estimated through leaf-clipping inoculation. The outcomes of our analysis showed that the bacterial blight symptoms and lesion lengths were significantly reduced in Δ*rpoN1*, Δ*rpoN2*, and Δ*rpoN1N2* than the wild-type strain. These diseased phenotypes were restored in the complemented strains (Figures 2A,B). Similarly, the Δ*rpoN1*, Δ*rpoN2*, and Δ*rpoN1N2* growth in rice leaf tissues were significantly reduced than the wild-type strain (Figure 2C). Thus, these results indicated that RpoN1 and RpoN2 positively regulated the virulence of *Xoo* in rice.

### RNA-Seq Analysis Identifies DEGs in *rpoN* Mutants

**FIGURE 3:**
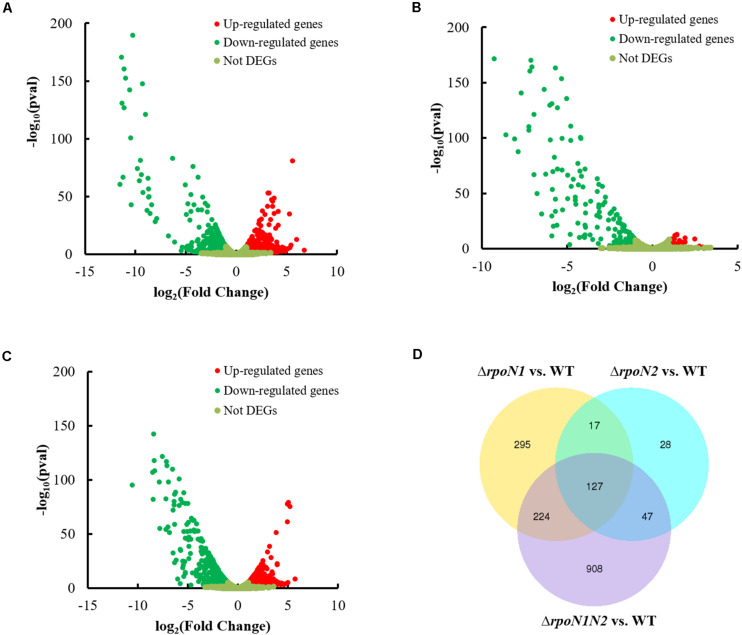
Differentially-expressed genes (DEGs) in Δ*rpoN1*, Δ*rpoN2*, and Δ*rpoN1N2*. DEGs in **(A)** Δ*rpoN1*, **(B)** Δ*rpoN2*, and **(C)** Δ*rpoN1N2*. Red and green dots represent up- and down-regulated genes, respectively. **(D)** RpoN1 and RpoN2 regulated unique and overlapping DEGs. Data represent the number of DEGs. DEGs at each mutant were selected according to the standards below: the absolute value of log_2_(Fold Change) > 1 and adjusted *P*-value < 0.05.

To apprehend RpoN1 and RpoN2 regulated motility and virulence of *Xoo*, RNA-Seq analysis of the *Xoo* wild-type and *rpoN* mutant strains was performed. DEGs at each mutant were identified as per the standards below: the absolute value of log_2_ (Fold Change) > 1 and adjusted *P*-value < 0.05. A total of 338 up-regulated and 325 down-regulated DEGs were identified in Δ*rpoN1* (Figure 3A), and in the Δ*rpoN2*, 49 up-regulated, and 170 down-regulated DEGs were identified as compared to wild-type strain (Figure 3B). Besides, 575 up-regulated and 731 down-regulated DEGs were identified in the double mutant Δ*rpoN1N2* compared to the wild-type strain (Figure 3C). Additionally, the Venn diagram was employed to analyze transcriptomic profiles, which showed 127 overlapping DEGs in different mutant strains (Figure 3D). To validate the reliability of RNA-seq results, the expression levels of 40 randomly selected DEGs were analyzed using the qRT-PCR assay. The results indicated that the gene expression patterns were in line with the RNA-seq analysis, indicating the reliability of RNA-seq results (Supplementary Figure 2).

### GO and KEGG Enrichment Analysis of the DEGs

**FIGURE 4:**
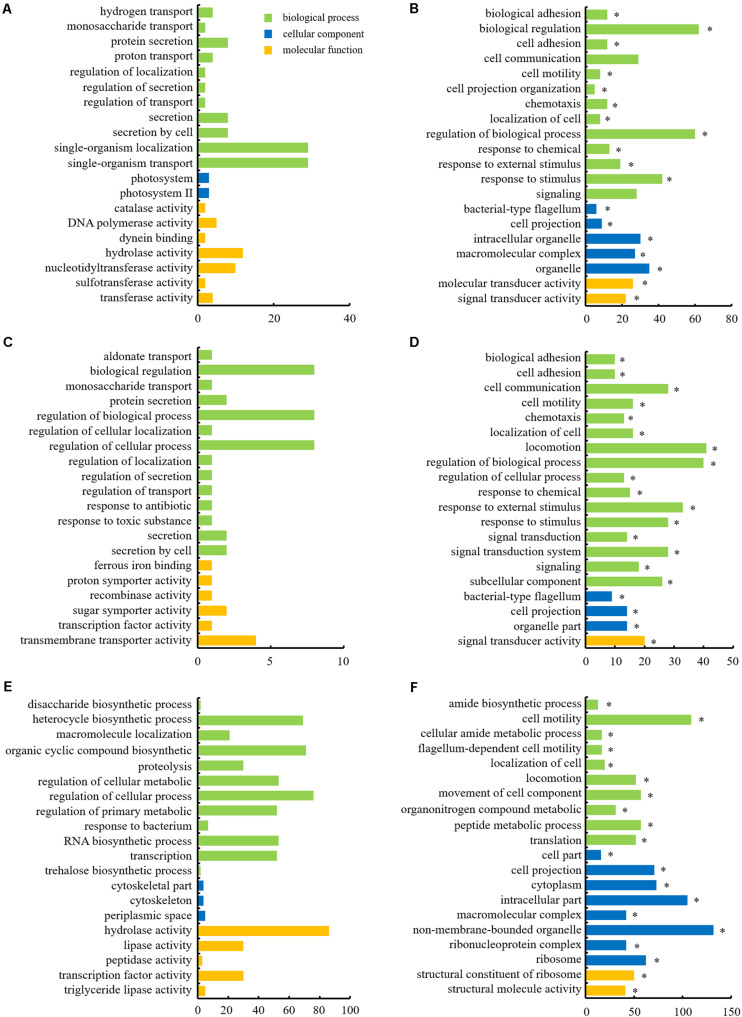
Histograms depicting GO analysis of up- and down-regulated genes in mutants. **(A)** Up-regulated and **(B)** down-regulated genes in Δ*rpoN1*. **(C)** Up-regulated and **(D)** down-regulated genes in Δ*rpoN2*. **(E)** Up-regulated and **(F)** down-regulated genes in Δ*rpoN1N2*. The abscissa axis represents the number of DEGs, and the ordinate axis represents the GO category. Asterisks indicate significant enrichment (*P* < 0.05).

The DEGs were categorized into three functional groups: biological process, cellular component, and molecular function, as per the GO enrichment analysis. The majority of the DEGs in Δ*rpoN1*, Δ*rpoN2*, and Δ*rpoN1N2* were enriched in the biological process category (Figure 4). In the biological process category, up-regulated genes were mostly enriched in single-organism localization and transport, biological regulation, and regulation of the cellular process, whereas down-regulated genes were mostly enriched in biological regulation, locomotion, and cell motility in Δ*rpoN1*, Δ*rpoN2*, and Δ*rpoN1N2* (Figure 4). As speculated, most down-regulated genes associated with bacterial motility were significantly enriched in the GO database’s biological process category (Figures 4B,D,F). In the cellular component category, down-regulated genes in Δ*rpoN1N2* were highly enriched in ribosomal associated categories (Figure 4F). It indicated that RpoN1 and RpoN2 regulate DEG expression by influencing ribosomal functions.

**FIGURE 5:**
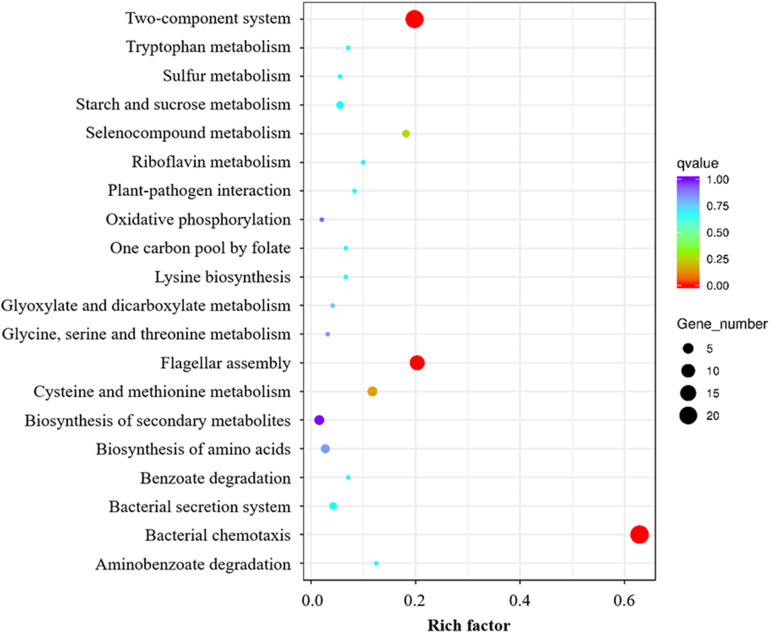
KEGG enrichment analysis of overlapping pathways regulated by both RpoN1 and RpoN2. Different colors represent the enriched pathways, and the size of the circle represents the number of DEGs.

To discern if RpoN1 and RpoN2 regulate *Xoo* motility and virulence through overlapping pathways, a total of 127 RpoN1 and RpoN2 regulated DEGs were analyzed using KEGG pathway analysis. These DEGs were significantly enriched in flagellar assembly, bacterial chemotaxis, and two-component system groups, suggesting the potential co-regulatory functions of RpoN1 and RpoN2 in *Xoo* (Figure 5).

### Flagellar Assembly Associated Genes Were Regulated by RpoN1 and RpoN2

**FIGURE 6:**
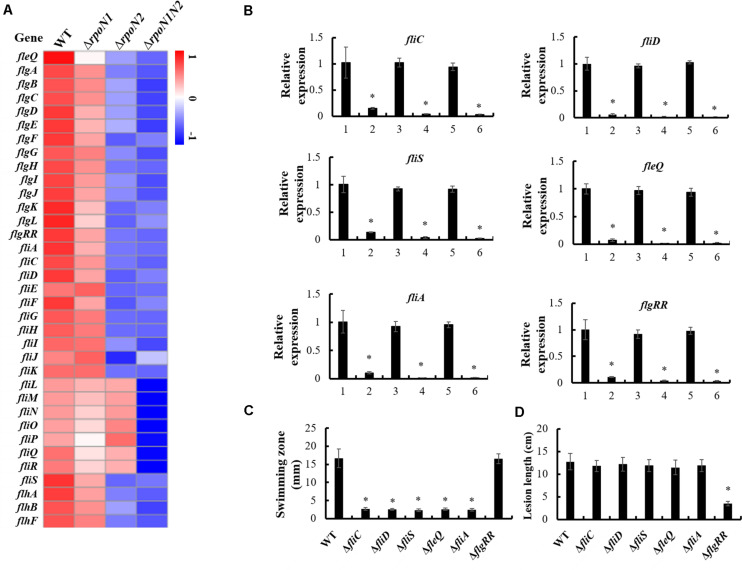
RpoN1 and RpoN2 regulated flagellar assembly. **(A)** Heat map of flagellar assembly associated genes expression. **(B)** The expression patterns of *fliC*, *fliD*, *fliS*, *fleQ*, *fliA*, and *flgRR* in Δ*rpoN1*, Δ*rpoN2*, and Δ*rpoN1N2* were validated using qRT-PCR. 1, WT; 2, Δ*rpoN1*; 3, Δ*rpoN1*-C; 4, Δ*rpoN2*; 5, Δ*rpoN2*-C; 6, Δ*rpoN1N2*. **(C)** Swimming motility was tested in Δ*fliC*, Δ*fliD*, Δ*fliS*, Δ*fleQ*, Δ*fliA*, and Δ*flgRR*. **(D)** Virulence was detected in Δ*fliC*, Δ*fliD*, Δ*fliS*, Δ*fleQ*, Δ*fliA*, and Δ*flgRR*. Asterisks indicate significant differences between WT and mutants (*P*-value < 0.05) (Student’s *t* test).

In our previous work, the gene cluster associated with flagellar assembly in *Xoo* was identified (Tian et al., 2015). In this study, 35 genes encoding flagellar structural components and regulatory factors in this gene cluster were down-regulated by RpoN1 and RpoN2 (Figure 6A). Most of these gene’s expression levels were significantly decreased in Δ*rpoN2* than Δ*rpoN1* (Figure 6A). It indicated that RpoN2 plays a more crucial regulatory role in flagellar assembly than RpoN1. Expression levels of three flagellar structural component genes, *fliC*, *fliD*, and *fliS*, and three regulatory factor genes, *fleQ*, *fliA*, and *flgRR* were validated using qRT-PCR. As per the outcomes of PCR based analysis, expression levels of these genes were down-regulated in Δ*rpoN1* and Δ*rpoN2* and restored in the relevant complemented strains (Figure 6B). Lastly, swimming motility and virulence of these gene deletion mutants were also determined. Interestingly, swimming zones were significantly decreased in Δ*fliC*, Δ*fliD*, Δ*fliS*, Δ*fleQ*, and Δ*fliA*, but not in Δ*flgRR* as compared to wild-type strain (Figure 6C). However, except for Δ*flgRR*, virulence between the wild-type strain and these mutants did not differ significantly, displaying reduced lesion length (Figure 6D).

### Chemotaxis Related Genes Were Down-Regulated by RpoN1 and RpoN2

**FIGURE 7:**
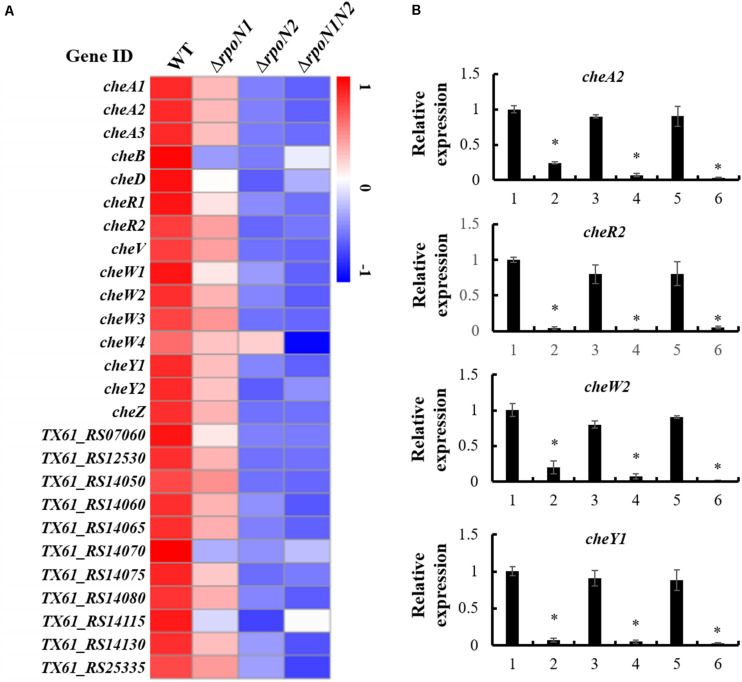
RpoN1 and RpoN2 regulated chemotaxis associated genes. **(A)** Heat map of gene expression of chemotaxis genes. **(B)** The expression patterns of *cheA2*, *cheR2*, *cheW2*, and *cheY1* in Δ*rpoN1*, Δ*rpoN2*, and Δ*rpoN1N2* were validated using qRT-PCR. 1, WT; 2, Δ*rpoN1*; 3, Δ*rpoN1*-C; 4, Δ*rpoN2*; 5, Δ*rpoN2*-C; 6, Δ*rpoN1N2*. Asterisks indicate significant differences between WT and mutants (*P*-value < 0.05) (Student’s *t* test).

Multiple DEGs were highly enriched in bacterial chemotaxis. Also, expression patterns of 26 genes encoding the core chemotaxis components, auxiliary proteins, and putative chemoreceptors in the RNA-seq data were specifically inspected. Similar to the flagellar assembly genes, RpoN1 and RpoN2 down-regulated the chemotaxis genes, and their expression levels were remarkably reduced in Δ*rpoN2* than Δ*rpoN1* (Figure 7A). Also, the expression patterns of four core chemotaxis components genes, *cheA2*, *cheR2*, *cheW2*, and *cheY1*, were validated using qRT-PCR. As per the outcomes, transcription levels of these genes were significantly down-regulated in Δ*rpoN1* and Δ*rpoN2* and restored in the relevant complemented strains (Figure 7B).

### c-di-GMP Associated Genes Were Regulated by RpoN1 and RpoN2

**FIGURE 8:**
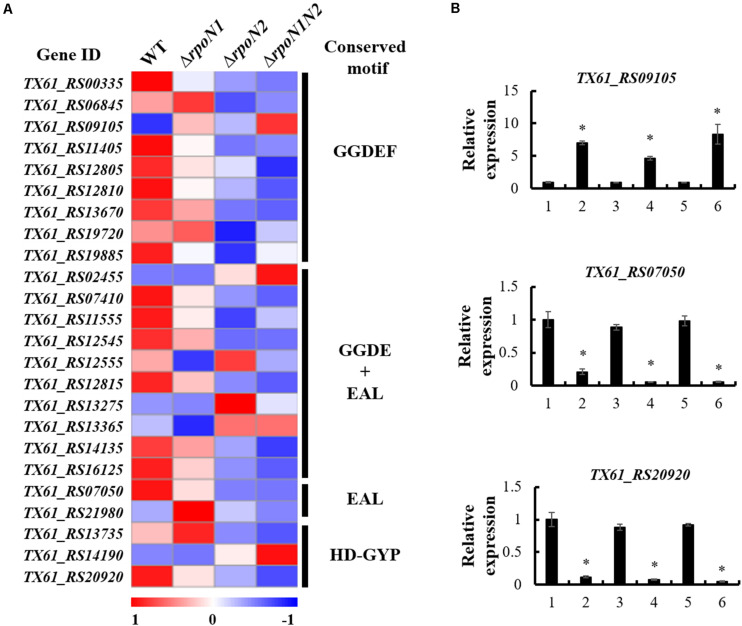
RpoN1 and RpoN2 regulated the expression of c-di-GMP associated genes. **(A)** Heat map of c-di-GMP associated gene expression. **(B)** The expression patterns of *TX61_RS09105*, *TX61_RS07050*, *TX61_RS20920* in Δ*rpoN1*, Δ*rpoN2*, and Δ*rpoN1N2* were validated by qRT-PCR. 1, WT; 2, Δ*rpoN1*; 3, Δ*rpoN1*-C; 4, Δ*rpoN2*; 5, Δ*rpoN2*-C; 6, Δ*rpoN1N2*. Asterisks indicate significant differences between WT and mutants (*P*-value < 0.05) (Student’s *t* test).

c-di-GMP, the widespread second messenger in pathogenic bacteria, plays a crucial role in regulating *Xoo* motility and virulence (Yang et al., 2019). Diguanylate cyclase (DGC) enzyme contains a conserved GGDEF domain, which regulates c-di-GMP synthesis, whereas phosphodiesterase (PDE) enzyme-containing conserved EAL or HD-GYP domains controls c-di-GMP degradation. To elucidate if RpoN1 and RpoN2 regulate the c-di-GMP synthesis or degradation in *Xoo*, the expression patterns of 24 genes encoding proteins with GGDEF, EAL, or HD-GYP domains in the RNA-seq data were analyzed. The outcomes indicated that six of nine genes encoding GGDEF domain-containing proteins, six of ten genes encoding GGDEF and EAL domains-containing proteins, one of two genes encoding EAL domain-containing proteins, and one of three genes encoding HD-DYP domain-containing proteins were down-regulated in Δ*rpoN1* and Δ*rpoN2* (Figure 8A). In addition, *TX61_RS09105*, the gene encoding GGDEF domain-containing protein, was up-regulated in Δ*rpoN1* and Δ*rpoN2* (Figure 8A). Three of these genes were selected and their expression patterns were validated using qRT-PCR based analysis (Figure 8B). It indicated that RpoN1 and RpoN2 might regulate the c-di-GMP synthesis and degradation by regulating DGC and PDE enzyme transcription in *Xoo*.

### RpoN1 Indirectly Regulates the *rpoN2* Expression

**FIGURE 9:**
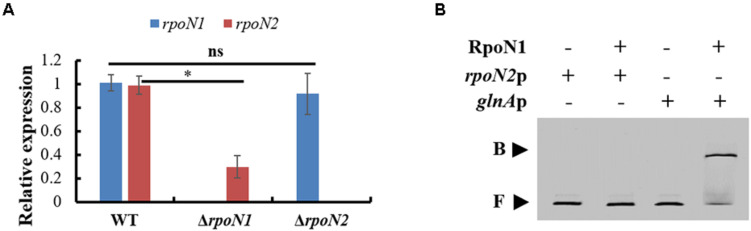
RpoN1 indirectly regulates the expression of *rpoN2*. **(A)** The expression patterns of *rpoN1* and *rpoN2* in Δ*rpoN1* and Δ*rpoN2* were investigated using qRT-PCR. **(B)** EMSA analysis to assess the interaction between RpoN1 and *rpoN2* promoter. *rpoN2*p, the promoter of *rpoN2*; *glnA*p, the promoter of *glnA*; B, binding probe; F, free probe. In RNA-seq data, the *glnA* encoded glutamine synthetase expression was significantly decreased in Δ*rpoN1* (data not shown). Thus, *glnA*p was selected as a positive control. Asterisk and ns indicate significant and insignificant differences, respectively (*P*-value < 0.05) (Student’s *t* test).

In the RNA-seq data, the *rpoN2* expression level was decreased in Δ*rpoN1*, but the *rpoN1* expression level did not differ significantly in Δ*rpoN2* (data not shown). These expression patterns were validated using qRT-PCR (Figure 9A). However, as per EMSA, direct binding of RpoN1 to the promoter of *rpoN2* was not observed (Figure 9B). It indicated that RpoN1 indirectly regulated the *rpoN2* expression in *Xoo*.

### RpoN1 and RpoN2 Affect the Growth of *Xoo*

**FIGURE 10:**
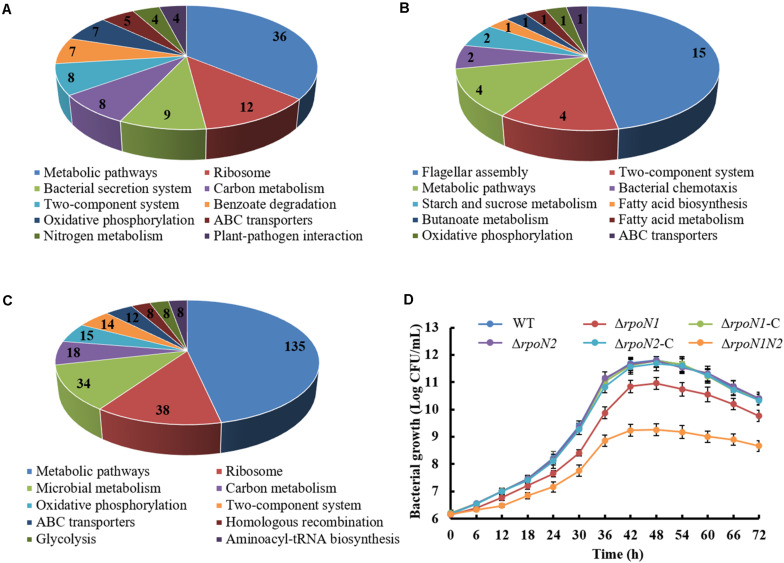
Analysis of DEGs specifically regulated by RpoN1 or RpoN2. DEGs specifically regulated by **(A)** *rpoN1*, **(B)** *rpoN2*, **(C)** Δ*rpoN1N2.* The number represents the number of DEGs enriched in KEGG pathways. **(D)** Growth curves of *Xoo* strain cultured using rich media M210. The growth curves were constructed three times, independently. Error bars indicate standard deviation.

To apprehend the distinct roles of RpoN1 and RpoN2 in regulating various biological pathways in *Xoo*, RpoN1, or RpoN2 regulated DEGs were analyzed using KEGG. The top ten KEGG pathways enriching these DEGs were recorded. It showed that majority of RpoN1 regulated DEGs were enriched in metabolic pathways, followed by the ribosome (Figure 10A), whereas the majority of RpoN2 regulated DEGs were enriched in flagellar assembly followed by the two-component system (Figure 10B). Interestingly, the majority of both RpoN1 and RpoN2 regulated DEGs were enriched in metabolic pathways, followed by the ribosome and microbial metabolism (Figure 10C). The bacterial metabolic process is closely associated with bacterial growth. In this study, we assessed the growth curves of wild-type strain, Δ*rpoN1*, Δ*rpoN1*-C, Δ*rpoN2*, Δ*rpoN2*-C, and Δ*rpoN1N2* strains. As the result showed, Δ*rpoN1*significantly reduced bacterial growth, and Δ*rpoN1N2* showed more serious inhibition of bacterial growth than Δ*rpoN1*, whereas Δ*rpoN2*, Δ*rpoN1*-C, Δ*rpoN2*-C, and wild-type bacterial strain showed similar growth curves (Figure 10D). These results suggested that the function of RpoN2 in *Xoo* growth might be complemented by RpoN1.

## Discussion

σ^54^ factors control a myriad of crucial biological functions in bacteria, such as motility, nitrogen metabolism, stress resistance, and virulence factor production (Dasgupta et al., 2003; Kazmierczak et al., 2005; Viducic et al., 2017). In *Xoo*, two σ^54^ factors, RpoN1 and RpoN2, were identified, whereas their overlapping or unique regulatory roles remain unknown (Tian et al., 2015). In this study, RNA-seq was employed to analyze the transcriptome-wide regulome of RpoN1 and RpoN2 and elucidate their co-regulatory and specific-regulatory pathways in *Xoo*. In previous reports, the regulatory roles of two homologous σ^54^ factors were found to be no overlapping in *Xanthomonas campestris* (Li et al., 2020). However, in *Xoo*, both RpoN1 and RpoN2 were involved in regulating flagellar assembly, chemotaxis and c-di-GMP synthesis, and degradation. In addition, the function of RpoN2 in *Xoo* growth might be complemented by RpoN1, and the *rpoN2* transcription level was indirectly regulated by RpoN1, while the *rpoN1* expression was not regulated by RpoN2, indicating the different regulatory roles of RpoN1 and RpoN2 in *Xoo*. Thus, these findings could extend our understanding of the overlapping and unique regulatory roles of σ^54^ factor homologous in bacteria.

Flagellar assembly is a highly organized process requiring the temporal expression of multiple genes, regulated in a hierarchical manner. In *P. aeruginosa*, a four-tiered transcriptional cascade including σ^54^-dependent transcriptional activator FleQ (class I), two-component system FleSR (class II), σ^28^ factor FliA (class III), and flagellin gene *fliC* (class IV) was identified (Dasgupta et al., 2003). Our previous report showed that RpoN2/FleQ regulated the *fliC* gene by regulating the transcription of *fliA* in *Xoo* (Tian et al., 2015). In this work, more than thirty flagellar-related genes, including flagellar structural component genes *fliC*, *fliD*, and *fliS*, and regulatory factor genes *fleQ*, *fliA*, and *flgRR* that were positively regulated by RpoN1 and RpoN2 were identified (Figures 6A,B). The innate immune response triggering *fliC* gene-encoded flagellin protein has been well studied as a pathogen-associated molecular pattern in *Arabidopsis* (Chinchilla et al., 2006). The *fliC* gene deletion blocked swimming motility, but it did not affect the *Xoo* pathogenicity in rice significantly (Figures 6C,D; Wang et al., 2015). Impaired swimming motility and similar pathogenicity in rice were also found in Δ*fliD*, Δ*fliS*, Δ*fleQ*, and Δ*fliA* mutants as compared to wild-type strain (Figures 6C,D). It indicated that RpoN1 and RpoN2 controlled bacterial virulence in a manner different from their regulatory role in flagellar motility of *Xoo*. In *X. citri*, the response regulator VemR, the homologous protein of FlgRR, functions as a RpoN2 cognate activator to positively regulate motility and virulence (Wu et al., 2019). In this study, RpoN1 and RpoN2 regulated *flgRR* expression, and *flgRR* deletion did not reduce *Xoo’s* swimming motility but remarkably decreased its virulence (Figure 6). The outcomes of this study suggested that RpoN1 and RpoN2 might regulate the bacterial swimming motility and virulence in *Xoo* by regulating the expression of flagellar synthesis genes and *flgRR*, respectively.

In bacteria, chemotaxis regulates directional motility, and thus it plays a crucial role in the adaptation of bacteria to changing environmental conditions. The basic chemotaxis mechanism of the signal transduction system was discerned in *E. coli* (Wadhams and Armitage, 2004; Parkinson et al., 2015). Briefly, activated chemoreceptor and phosphorylated cytoplasmic sensor kinase CheA recognizes the chemosensory signal. Subsequently, adaptor protein CheW mediates the transfer of phosphate radical of CheA to the response regulator CheY. Thus, CheY regulates flagellar motor activity. Meanwhile, methy-transferase CheR binds to CheA, CheW, and CheY and constitutes the core components of the chemotactic signal transduction pathway. A previous study also showed that in *Xoo*, core elements of the chemotaxis signal transduction system, including CheA2, CheR2, CheW2, and CheY1, entailed several paralogs of chemotaxis components (Kumar Verma et al., 2018). In addition, deficiency of these core components significantly mitigated virulence-associated functions, such as attachment and iron homeostasis (Kumar Verma et al., 2018). In this study, more than twenty RpoN1 and RpoN2 regulated chemotaxis components and putative chemoreceptors genes were identified (Figure 7A). The expression patterns of *cheA2*, *cheR2*, *cheW2*, and *cheY1* were validated using qRT-PCR in Δ*rpoN1* and Δ*rpoN2* (Figure 7B). The results of the current study indicated that RpoN1 and RpoN2 might regulate the virulence by regulating the chemotaxis pathway in *Xoo*.

c-di-GMP plays a vital role in regulating multiple biological functions, such as motility, biofilm formation, EPS production, environment adaptation, and virulence (Hengge, 2009). The antagonistic enzymatic actions of DGC and PDE enzymes regulate the synthesis and degradation of intracellular c-di-GMP, respectively. DGCs contain a conserved GGDEF domain, while PDEs contain conserved EAL or HD-GYP domains (Yang et al., 2019). A previous study reported that two GGDEF domain proteins, GdpX1 from *Xoo* strain PXO99^*A*^ and DgcA from *Xoo* strain KACC10331, negatively regulates bacterial virulence, EPS production, and motility (Su et al., 2016; Yang et al., 2016). EdpX1, an EAL domain protein, exhibited PDE activity, positive regulation of c-di-GMP level and virulence in *Xoo* strain PXO99^*A*^ (Xue et al., 2018). In this study, a putative DGC protein containing GGDEF domain encoded by *TX61_RS09105* was overexpressed in Δ*rpoN1* and Δ*rpoN2*. Besides, two putative PDEs containing EAL and HD-GYP domain encoded by *TX61_RS07050* and *TX61_RS20920*, respectively, were down-regulated in Δ*rpoN1* and Δ*rpoN2* (Figure 8A). The results suggested that RpoN1 and RpoN2 control the synthesis and degradation of c-di-GMP by regulating DGCs and PDEs in *Xoo*.

Although two or more *rpoN* copies were identified in certain bacteria, the difference in these RpoN proteins’ regulatory roles was rarely reported. In *R*. *solanacearum*, *rpoN1*, but not *rpoN2*, is necessary for virulence, twitching motility, natural transformation, and growth on nitrate (Ray et al., 2015). However, in *X*. *campestris*, RpoN1 regulates branched-chain fatty acid production and diffusible signal synthesis, whereas RpoN2 regulates swimming motility, biofilms, EPS production, and virulence (Li et al., 2020). Unlike these studies, in the current study, both RpoN1 and RpoN2 were involved in regulating *Xoo* swimming motility and virulence (Figures 1, 2). Interestingly, the transcription levels of flagellar assembly-related genes were substantially decreased in Δ*rpoN2* than Δ*rpoN1* (Figure 6A). Also, RpoN1 indirectly regulated the *rpoN2* expression (Figure 9). It indicated that in *Xoo*, RpoN1 might regulate motility by regulating *rpoN2* expression. Moreover, RpoN1 regulated multiple DEGs related to ribosome, carbon, and nitrogen metabolism whereas only a few DEGs related to metabolic pathways were specifically regulated by RpoN2 in *Xoo* (Figures 10A,B). Importantly, RpoN1 is more important than RpoN2 for *Xoo* growth in rich medium (Figure 10D). These results suggested that as compared to RpoN2, RpoN1 plays a more crucial role in regulating basal metabolism and growth in *Xoo*.

## Data Availability Statement

The datasets presented in this study can be found in online repositories. The names of the repository/repositories and accession number(s) can be found in the article/Supplementary Material.

## Author Contributions

CY and D-PN designed and performed the experiments and wrote the manuscript. FY, JS, and YW analyzed the data and commented on the manuscript. FT commented on the manuscript. XZ and HC designed the experiments, supervised the research, and finalized the manuscript. All authors contributed to the article and approved the submitted version.

## Conflict of Interest

The authors declare that the research was conducted in the absence of any commercial or financial relationships that could be construed as a potential conflict of interest.

## Footnotes

1 https://CRAN.R-project.org/package=gplots

## Supplementary Material

Supplementary Figure 1 Conserved σ^54^ factors in *P. syringae* pv. *tomato* DC3000 (*Pst*), *X. campestris* pv. *campestris* (*Xcc*), and *Xoo* strains. The σ^54^ factor sequences were downloaded from the National Center for Biotechnology Information database and aligned using DNAMAN based on identical residues. Activator interacting domain (AID) and DNA binding domain (DBD) were highly conserved among σ^54^ proteins from different species.

Supplementary Figure 2 Correlation analysis between qRT-PCR and RNA-seq data of forty selected DEGs. Analysis of the expression patterns of DEGs in **(A)** Δ*rpoN1*, **(B)** Δ*rpoN2*, and **(C)** Δ*rpoN1N2*.

Supplementary Table 1 Bacterial strains and plasmids used in this study.

Supplementary Table 2 Primers used in this study.

Supplementary Table 3 Expression patterns of selected DEGs in Δ*rpoN1*, Δ*rpoN2*, and Δ*rpoN1N2* from RNA-seq data.
