# Supplementary figures and images for: Transcriptome Analysis Revealed Overlapping and Special Regulatory Roles of RpoN1 and RpoN2 in Motility, Virulence, and Growth of Xanthomonas oryzae pv. oryzae
Source: Front Microbiol. 2021 Mar 4;12:653354. doi: 10.3389/fmicb.2021.653354 (PMC7970052; doi:10.3389/fmicb.2021.653354)

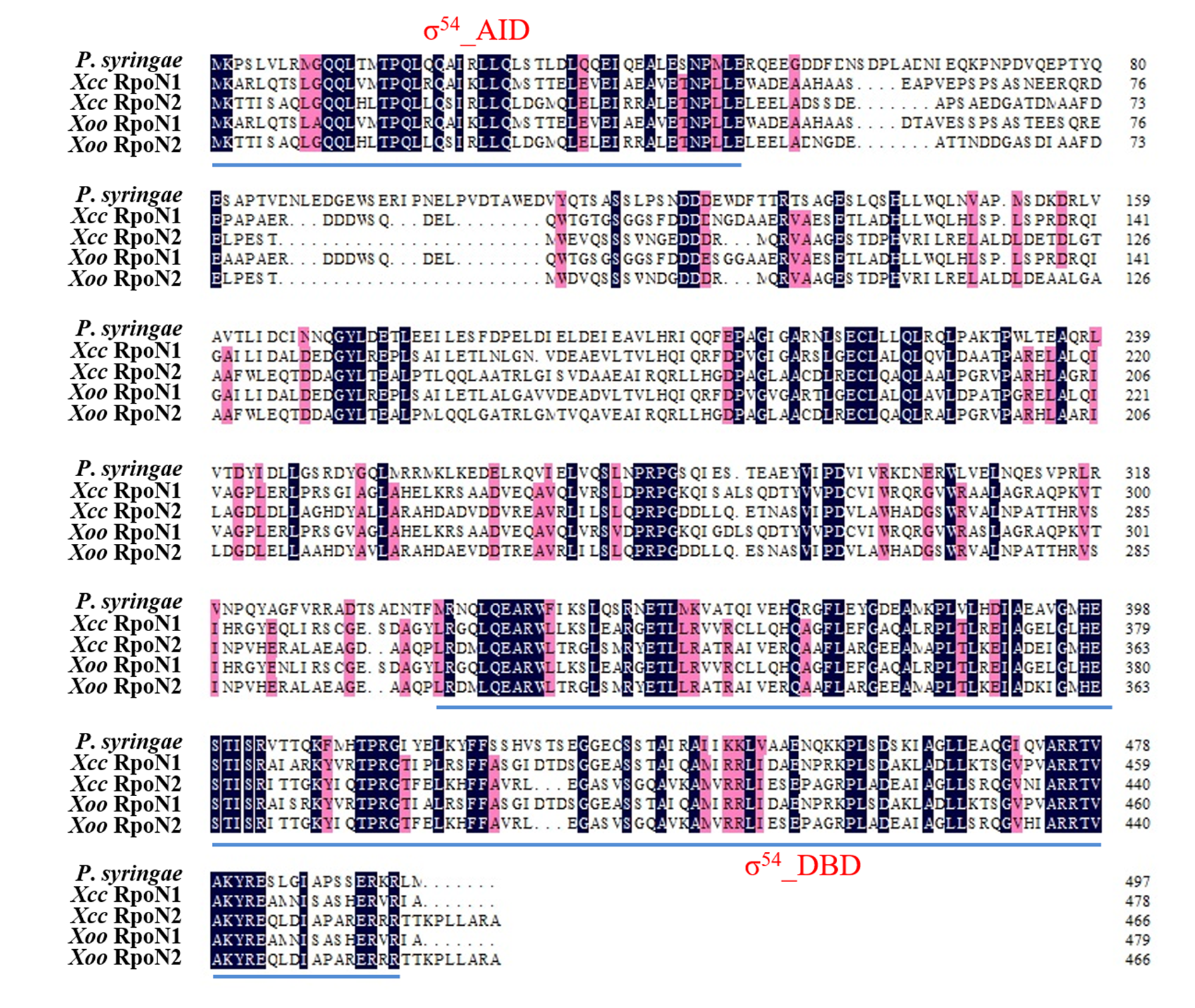

Supplement: Supplementary Figure 1 — Conserved σ54 factors in P. syringae pv. tomato DC3000 (Pst), X. campestris pv. campestris (Xcc), and Xoo strains. The σ54 factor sequences were downloaded from the National Center for Biotechnology Information database and aligned using DNAMAN based on identical residues. Activator interacting domain (AID) and DNA binding domain (DBD) were highly conserved among σ54 proteins from different species. [file Image_1.TIF]

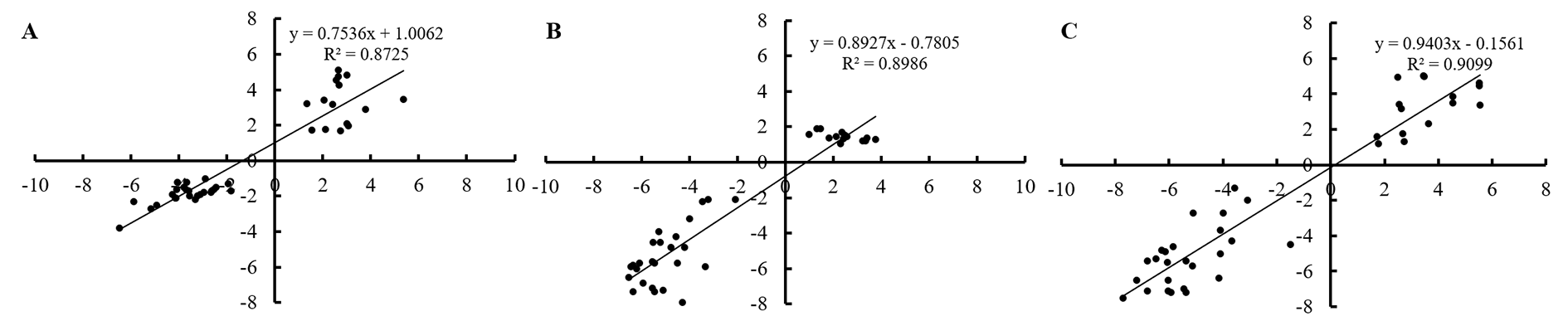

Supplement: Supplementary Figure 2 — Correlation analysis between qRT-PCR and RNA-seq data of forty selected DEGs. Analysis of the expression patterns of DEGs in (A) ΔrpoN1, (B) ΔrpoN2, and (C) ΔrpoN1N2. [file Image_2.TIF]
