# Supplementary material for: Transcriptome Analysis Revealed Overlapping and Special Regulatory Roles of RpoN1 and RpoN2 in Motility, Virulence, and Growth of Xanthomonas oryzae pv. oryzae
Source: Front Microbiol. 2021 Mar 4;12:653354. doi: 10.3389/fmicb.2021.653354 (PMC7970052; doi:10.3389/fmicb.2021.653354)
Supplement: Supplementary Table 1 — Bacterial strains and plasmids used in this study. [file Table_1.DOCX]

**Table S1. Bacterial strains and plasmids used in this study**

| **Strain or plasmid** | **Relevant characteristics^a^** | **Reference or source** |
| --- | --- | --- |
| ***Escherichia coli*** |  |  |
| DH5α | *supE44* Δ*lacU169(Φ80lacZ*Δ*M15) hsdR17 recA1 endA1 gyrA96 thi-1 relA1* | Hanahan 1983 |
| BL21 | For protein expression | Novagen |
| ***Xanthomonas oryzae* pv. *oryzae*** |  |  |
| MAFF 311018 | Wild-type strain | Lab collection |
| Δ*rpoN1* | *rpoN1* gene deletion mutant | This study |
| Δ*rpoN2* | *rpoN2* gene deletion mutant | Our lab |
| Δ*rpoN1N2* | *rpoN1* and *rpoN2* double mutant | This study |
| Δ*fliC* | *fliC* gene deletion mutant | This study |
| Δ*fliD* | *fliD* gene deletion mutant | This study |
| Δ*fliS* | *fliS* gene deletion mutant | This study |
| Δ*fleQ* | *fleQ* gene deletion mutant | This study |
| Δ*fliA* | *fliA* gene deletion mutant | This study |
| Δ*flgRR* | *flgRR* gene deletion mutant | This study |
| Δ*rpoN1*-C | Δ*rpoN1* containing plasmid pBBR-*rpoN1*, Ap^r^ | This study |
| Δ*rpoN2*-C | Δ*rpoN2* containing plasmid pBBR-*rpoN2*, Ap^r^ | This study |
| **Plasmid** |  |  |
| pKMS1 | Suicidal vector carrying *sacB* gene for non-marker mutagenesis, Km^r^ | Li *et al*., 2011 |
| pKM-*rpoN1* | pKMS1 derivative carrying a *rpoN1* mutation, Km^r^ | This study |
| pKM-*fliC* | pKMS1 derivative carrying a *fliC* mutation, Km^r^ | This study |
| pKM-*fliD* | pKMS1 derivative carrying a *fliD* mutation, Km^r^ | This study |
| pKM-*fliS* | pKMS1 derivative carrying a *fliS* mutation, Km^r^ | This study |
| pKM-*fleQ* | pKMS1 derivative carrying a *fleQ* mutation, Km^r^ | This study |
| pKM-*fliA* | pKMS1 derivative carrying a *fliA* mutation, Km^r^ | This study |
| pKM-*flgRR* | pKMS1 derivative carrying a *flgRR* mutation, Km^r^ | This study |
| pBBR1MCS-4 | Broad-host range expression vector, Ap^r^ | Kovach et al., 1995 |
| pBBR-*rpoN1* | pBBR1MCS-4 carrying the full length of *rpoN1*, Ap^r^ | This study |
| pBBR-*rpoN2* | pBBR1MCS-4 carrying the full length of *rpoN2* Ap^r^ | This study |
| pColdSUMO | Expression vector to generate a N-terminal SUMO-His_6_ tag, Ap^r^ | Haigene |
| pC-*rpoN1* | pColdSUMO carrying the coding sequence for RpoN1, Ap^r^ | This study |

^a^ Ap^r^ and Km^r^ indicate resistant to ampicillin and kanamycin, respectively.
