# Supplementary material for: Transcriptome Analysis Revealed Overlapping and Special Regulatory Roles of RpoN1 and RpoN2 in Motility, Virulence, and Growth of Xanthomonas oryzae pv. oryzae
Source: Front Microbiol. 2021 Mar 4;12:653354. doi: 10.3389/fmicb.2021.653354 (PMC7970052; doi:10.3389/fmicb.2021.653354)
Supplement: Supplementary Table 2 — Primers used in this study. [file Table_2.DOCX]

**Table S2 Primers used in this study**

| **Name** | **Sense primer (F)** | **Anti-sense primer (R)** | **Used for** |
| --- | --- | --- | --- |
| *rpoN1L* | CGGGATCCACACCAAGACCGGCAACATGCAAAG | CCAAGCTTGCGGGCACTGACTGAAAAAAGCGTT | mutant construction |
| *rpoN1R* | CCAAGCTTCGTTGCAACCACCGCAACGCCGGCC | GCTCTAGACTCGCGTGCGGTGATGCTGGTATTC | mutant construction |
| *fliCL* | CGGAATTCGTGTTTTCCACCATCGAC | CGGGATCCTACGTTGGTGTTGATTAC | mutant construction |
| *fliCR* | GCTCTAGACTTGCAGTCAGCTGCCGC | CCAAGCTTCATCGTTGGCGGTCACAA | mutant construction |
| *fliDL* | CGGGATCCATCGCCGACGCCAACAT | CCAAGCTTGTTGCATTCCTCGTCTGGGCGTC | mutant construction |
| *fliDR* | CCAAGCTTTTTGCGTGATGAATATCCTC | GCTCTAGACTAAGCGTGTCGGCAAAC | mutant construction |
| *fliSL* | CGGGATCCCAGTTACGACGCCACCACCAA | CCAAGCTTGGATGACTCCTCAATGGGCTGA | mutant construction |
| *fliSR* | CCAAGCTTGTCCTGTCATGCATAGCGTG | GCTCTAGATTCCTCGCTACGCAAGTC | mutant construction |
| *fleQL* | CGGGATCCGCTACGAGACCCTGCTGCGTGCAAC | CCAAGCTTTCATTCCTGGCTCCTTACCGTCAGG | mutant construction |
| *fleQR* | CCAAGCTTGCGGTCACACGCTCAGCTTGCGCTC | GCTCTAGACAACACCGGAAAATACGCGTAATTG | mutant construction |
| *fliAL* | CGGGATCCCTCGTTCTTCGCCTTGAAGATTTG | CCAAGCTTGCGGCAACCCCCCGTTGGATGATG | mutant construction |
| *fliAR* | CCAAGCTTTTTTTCGCGTATTTTTTCCTGTTAG | GCTCTAGACGACAGCTCGGTGAGGTTGTG | mutant construction |
| *flgRRL* | CGGGATCCTCTGGGTATGACCGTGCAGGCCGTG | CCAAGCTTAGTAGTCCTTATCCTGCCCGGGCAA | mutant construction |
| *flgRRR* | CCAAGCTTGTGAGTCCCGCATTCTGTTGATCGA | GCTCTAGACAGCTTGACCTGCATCGGCAGGCTC | mutant construction |
| *rpoN1* | CGGGATCCATGAAAGCTCGGCTCCAGACATCGC | GCTCTAGATCAGTGATGGTGATGGTGATGGGCGATGCGGACGCGTTCGTGG | complementary strain construction |
| *rpoN2* | CGGGATCCATGAAGACGACCATTTCCGCCCAGC | GCTCTAGATTAGTGATGGTGATGGTGATGTCCTGCCCGGGCAAGCAGCGGT | complementary strain construction |
| N1 | CGGGATCCATGAAAGCTCGGCTCCAGACAT | CCAAGCTTGGCGATGCGGACGCGTTCGTG | protein expression |
| *rpoN2p* | CTTCCAAACATCACAACGAGTTGGT | GGATCCTGAGGTGCAGTTGCTGG | EMSA |
| *glnAp* | GAAATCAGATCGGACATCGGAC | CGACCTTGTTGTCCTTGATC | EMSA |
| *TX61_RS00335* | CGGCATTCCCGACCTCAA | CGTTGTTCGCAATCTTCCACC | qRT-PCR |
| *TX61_RS00450* | TCGGTATCCGCACCCTCA | AACAGCAGCACGCCCAGT 3' | qRT-PCR |
| *TX61_RS00460* | CTTCCCTGCATGGTGTTGC | AATGGCGGGTCCGTTTGT 3' | qRT-PCR |
| *TX61_RS00465* | ACGCCAATGCGGCTTCGT | CGTCGTCGTCCTCGTTCAATAA 3' | qRT-PCR |
| *TX61_RS01580* | AGCCAACAGCCGACTTCATT | CAGCGATTGCCCATCCATT 3' | qRT-PCR |
| *TX61_RS02605* | AGCCTGCCGATCCGAAACCC | ACCTGGCCGCTCCACGAAA 3' | qRT-PCR |
| *TX61_RS04040* | GCAGCGAACACGGCATCA | CCAGCAGGTGGCGGACAAT 3' | qRT-PCR |
| *TX61_RS04045* | CGATGAGGTCCAGTTGTGCG | GGCGATGGTGTCCAGGGTT 3' | qRT-PCR |
| *TX61_RS04050* | TGCTGCCGCTGGAAGTGT | TCCTCGGAGAAGCCTGTGC 3' | qRT-PCR |
| *TX61_RS04055* | CTGGAGGGTGATTCCTGCTAT | CACCGTTCACGCCTTTGAT | qRT-PCR |
| *TX61_RS04060* | TGAAGGTGGAGCGGGTGTC | ATCGGCTTGAGGACCTGGAT | qRT-PCR |
| *TX61_RS04095* | AGGTCGCCCGCTGGTTGCTGAT | AACGCACTGTGCCGCCTGGTTC | qRT-PCR |
| *TX61_RS06110*  (*rpoN1*) | GCAGCCCAAGGTCACCATCCA | TGCACCGAACTCCAGGAACCC | qRT-PCR |
| *TX61_RS06555* | ATTGCGTTTGCTGTCGTGC | AAAGTGGGTCAGTGCCTTGC | qRT-PCR |
| *TX61_RS07050* | GCATTGACCCAGCAGGTGATC | CTCAGTTTGGCGCAGAGTTCTT | qRT-PCR |
| *TX61_RS07410* | CATGGAGATCACCGAGACCG | TTGCTTGAGATAACCGAACGAG | qRT-PCR |
| *TX61_RS08570* | AGTCGCGTCGGGAAAGGA | TGGTGCTCACAAAGTGCTGGTA | qRT-PCR |
| *TX61_RS09105* | GCGGCAGTGGGCGAATTGTTG | GCCTCGGGCAGAAGTACGGTGAA | qRT-PCR |
| *TX61_RS11555* | CTCGGACGAAGTGATGGTGG | CGACATAGAAGCGAATGAATGG | qRT-PCR |
| *TX61_RS12530* | GCCGCTCCTTTGGCTCTGA | GGAACTGGGAATACGTCACCC | qRT-PCR |
| *TX61_RS12545* | CGGCTTGTGGGACTGGGATA | GGCGGCTGCTCGGAATAA | qRT-PCR |
| *TX61_RS12570* | GCAGCAAGATCGCTTCGG | CATCCTTCACCGCCTGGAC | qRT-PCR |
| *TX61_RS12580* | GTGCCGAAGCCACTTATTTGA | ACCAGTTGCAGCGAGGGAT | qRT-PCR |
| *TX61_RS12625* | GGCAATCTGAGTGGCATCGG | CATCGGTCCAGCGGTCGTT | qRT-PCR |
| *TX61_RS12640* (*fliC*) | GAGCGCAACCTCGGAAAACC | GGAACCGACTTGGCCTGGG | qRT-PCR |
| *TX61_RS12645*  (*fliD*) | AAGGATGGCGTGATGAGTTTCG | CGTGCCGTTGGTTGCATTGAC | qRT-PCR |
| *TX61_RS12650*  (*fliS*) | GGTGGCGAGATCGCTGGTA | CACGAAACGGCGGAAAGG | qRT-PCR |
| *TX61_RS12660* | GTTTGCCGACACGCTTAGTTG | CGGTCTTCGCTGGTTTCCTC | qRT-PCR |
| *TX61_RS12670*  (*rpoN2*) | TGACTCGCGGGCTGTCGA | GCGGGGTCTGGATGTATTTGC | qRT-PCR |
| *TX61_RS12675*  (*flgRR*) | ACGAGGGCTTCATCAACG | CCAGGTCCATCAGCACGA | qRT-PCR |
| *TX61_RS12680*  (*fleQ*) | GGCCGTTCGTCGCAATCAA | GCAGGCTCATGTCGCCGATT | qRT-PCR |
| *TX61_RS12810* | CCACCTCCAATGGCTTGTTCC | CCTCACCGCTGTCGCTGATA | qRT-PCR |
| *TX61_RS12840*  (*fliA*) | CCCAACCCGCAGCAGATGA | CTGGCCGTGGATCTGGCAG | qRT-PCR |
| *TX61_RS12845*  (*cheY1*) | GGTAAGGAGCCAGGAATGAA | GGTATTGGTGAAGCCGAGAT | qRT-PCR |
| *TX61_RS12850* | AAATGCGCCACAACCTCACC | CCGCCGTCGTCTTTCTTCG | qRT-PCR |
| *TX61_RS12855*  (*cheA2*) | ATCGTCGAGGCCCAGGAAA | TCGGCTTGATCGCCAGAAA | qRT-PCR |
| *TX61_RS14000* | CGATGGTGGGCTATGGAGAA | GCCGTCAGACTTGGGAAGC | qRT-PCR |
| *TX61_RS14010*  (*cheW2*) | GCGTCCATCTGGGCTCTTC | TGATCGGTCAGGCTGGTGA | qRT-PCR |
| *TX61_RS14020* | AACATGGACGGCATCTCGC | TTCTGGATTGAACGGCTTGA | qRT-PCR |
| *TX61_RS14050* | CGACCCAGACCTCACCATCA | CGTCCTGTGCATTGCCTACTT | qRT-PCR |
| *TX61_RS14065* | AAAAGCAGCAACACCGAACTG | TGCAATGTCCCGCTACGC | qRT-PCR |
| *TX61_RS14080* | CCAGGCATTGCCGTTACTACTG | GCTTGCGTGAATCGTCCATC | qRT-PCR |
| *TX61_RS14085* | CTGGAAGTGGACGAAGACGA | TGGGTGTAGCACAGCAGGTAT | qRT-PCR |
| *TX61_RS14120*  (*cheR2*) | GCAACGACGAAGAAGAATGG | TGATGGCGATGGAGTAGGG | qRT-PCR |
| *TX61_RS18510* | GTGGTCGGGCGGTTGTATC | ATTGGCGAACAGCGGGTGG | qRT-PCR |
| *TX61_RS18520* | CGAACGCTTTCCGGTTGC | TGGTCGATGGGTTTGTCCTG | qRT-PCR |
| *TX61_RS20920* | AGACCGCCGACGACTACACC | CGACCGGGCTTGTTGAGAAT | qRT-PCR |
| *gyrb* | GGCGAGCACAATGGCATT | CCATCCTTCTGCGGGATGT | qRT-PCR |
